# Radial Artery Aneurysm Arising from the Stump of a Ligated Arteriovenous Fistula in a Kidney Transplant Patient

**DOI:** 10.1016/j.jpra.2022.08.004

**Published:** 2022-09-06

**Authors:** Amenah Dhannoon, Fiachra Sheil, Seamus McHugh, J. Barry O'Sullivan

**Affiliations:** a Royal College of Surgeons in Ireland, Dublin; b Department of Reconstructive and Plastic Surgery, Beaumont University Hospital, Dublin; c Department of Vascular Surgery, Beaumont University Hospital, Dublin

**Keywords:** Arteriovenous fistula, Radiocephalic arteriovenous fistula, Radial artery aneurysm, Management, Pathogenesis

## Abstract

While aneurysm and pseudoaneurysm are common complications to arteriovenous (AV) fistula, the development of aneurysm from the stump of a ligated AV fistula is unusual. The involvement of radial artery after AV fistula ligation is an extremely rare entity with only two cases reported in the literature. In this report, we describe a 40-year-old kidney transplant patient who presented with a radial artery aneurysm after radiocephalic fistula ligation which was managed by the plastic surgery team using autologous vein graft reconstruction, and we compare our case to the two cases described in the literature in the presentations, timelines, and management options.

## Introduction

Arteriovenous (AV) fistula is the gold standard vascular access for hemodialysis. The ligation of the AV fistula is recommended after transplantation to avoid local complications, such as hemorrhage, aneurysm, pseudoaneurysm, infection, thrombosis, and neuropathy, and systematic complications, such as steal syndrome, cardiac failure, and pulmonary hypertension.^1^ While aneurysm and pseudoaneurysm are common complications to AV fistula, the development of aneurysm from the stump of a ligated AV fistula is a rare entity. Several case reports have been published describing this entity in the brachial artery.^1^, ^2^, ^3^, ^4^, ^5^

Aneurysm is defined as 50% increase in the diameter of a vessel. The mean radial artery diameter in the Western population is 3.67 ± 0.8 mm.^6^ The incidence of non-traumatic true radial artery is rare with only 9 cases reported in the literature.^7^ Secondary aneurysm is commonly caused by iatrogenic, traumatic, and infection.^6^^,^^7^

In this case report, we highlight a true radial artery aneurysm arising following radiocephalic fistula ligation to the plastic surgeon's community and summarize the various presentations, timelines, investigations, and management options available in dealing with similar cases.

## Case Presentation

A 40-year-old gentleman presented to the vascular surgery clinic with a swelling over the palmar aspect of the left wrist. He reported the swelling has been there for three months and has been increasing in size causing him discomfort and cold intolerance in the left wrist. He had a history of end-stage kidney disease secondary to malignant hypertension. Ten years prior he had a radiocephalic fistula formation for hemodialysis. He then received a deceased kidney transplant five years later. The fistula was then surgically ligated six years after the transplant to avoid AV fistula complications. His medications include steroid (prednisolone 5 mg/day), tacrolimus (Prograf 10 mg/day), mycophenolate (Mycolate 250 mg/day), co-trimoxazole 480 mg/day, valganciclovir 450 mg/day, omeprazole 40 mg/day, amlodipine 5 mg/day, and ramipril 10 mg/day, in addition to calcium supplements.

**Fig. 1:**
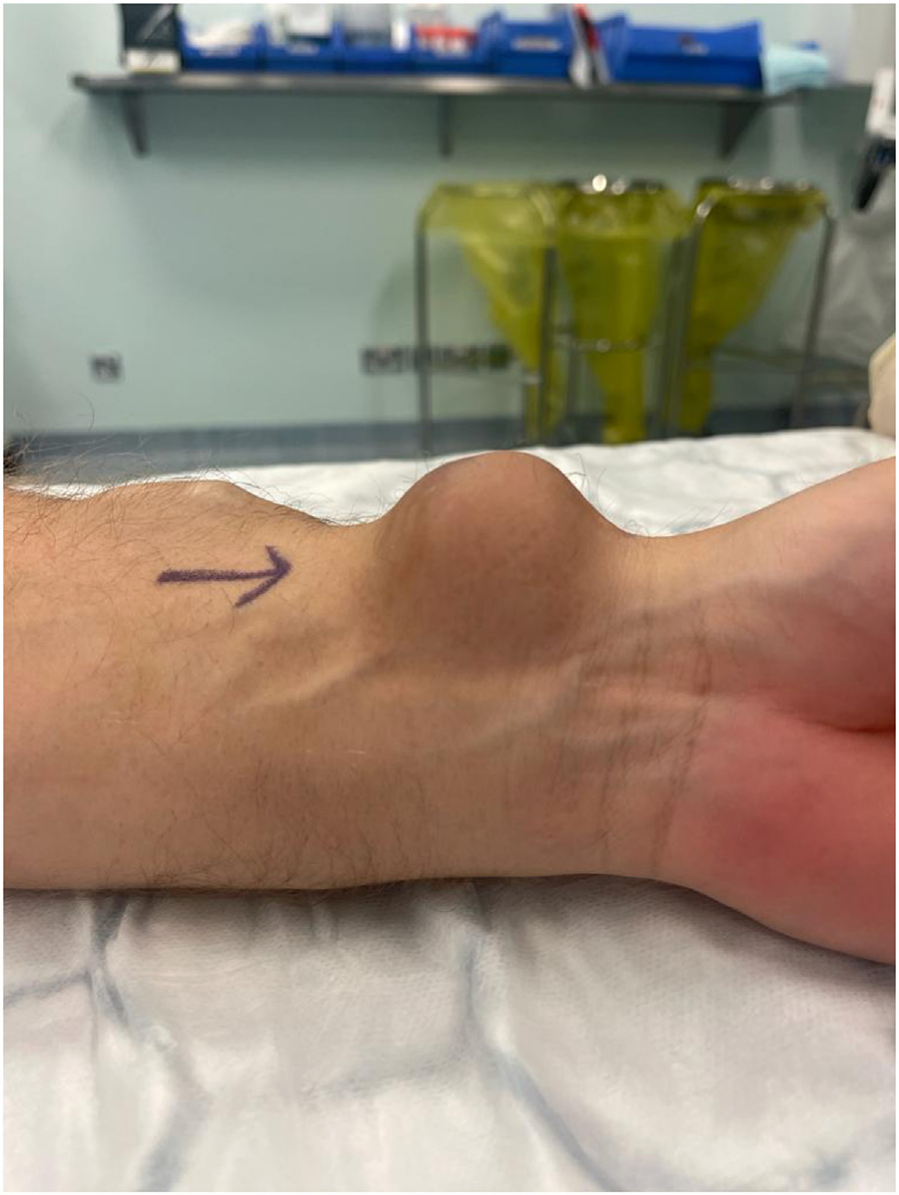
Preoperative image showing a 4x4 cm swelling at the volar aspect of the left wrist.

**Fig. 2:**
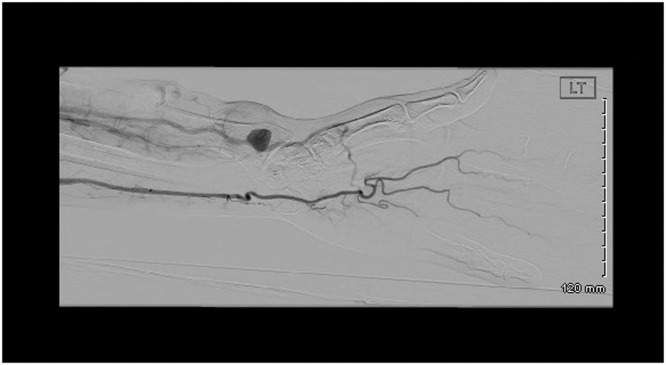
CT angiogram showing an aneurysmal dilatation of the radial artery and poor perfusion of the radial palmar arch with intact ulnar palmar arch.

On examination, the patient was hemodynamically stable. There was a pulsatile, non-tender lump of 4 × 3 cm in size, with normal overlying skin. The radial artery and ulnar artery were palpable. The left hand was cooler compared to the right hand. There was no neuromotor or neurosensory deficit (Fig. 1). Laboratory test results included hemoglobin 13.2 gm/dl, white cell count 6.14 10^9/L, Na 142 mmol/L, K 4.5 mmol/L, Urea 8.3 mmol/L, creatinine 165 umol/L, and tacrolimus level 6.8 ng/mL. He underwent a duplex ultrasound, which demonstrated an aneurysm of the radial artery with signs of turbulent blood flow represented in the radiological ying/yang signs and a thrombus within the aneurysm. He further had a computed tomography (CT) angiogram to confirm the patency of the palmar arch. This the ulnar arch was patent; however, there was a poor perfusion of the thumb and radial side of the index (Fig. 2).

**Fig. 3:**
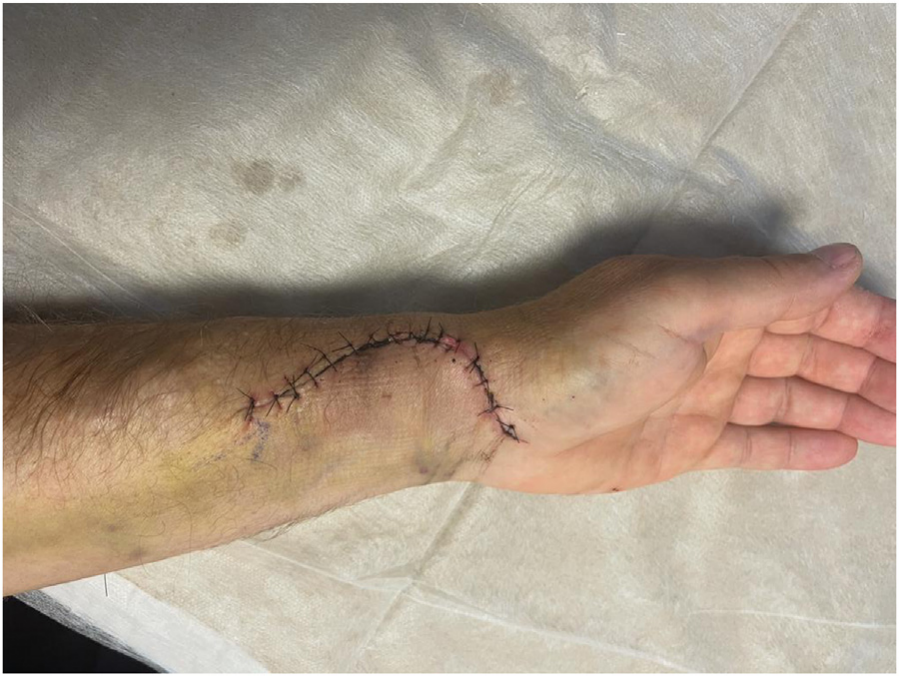
Postoperative image after the surgical excision of the aneurysm.

**Fig. 4:**
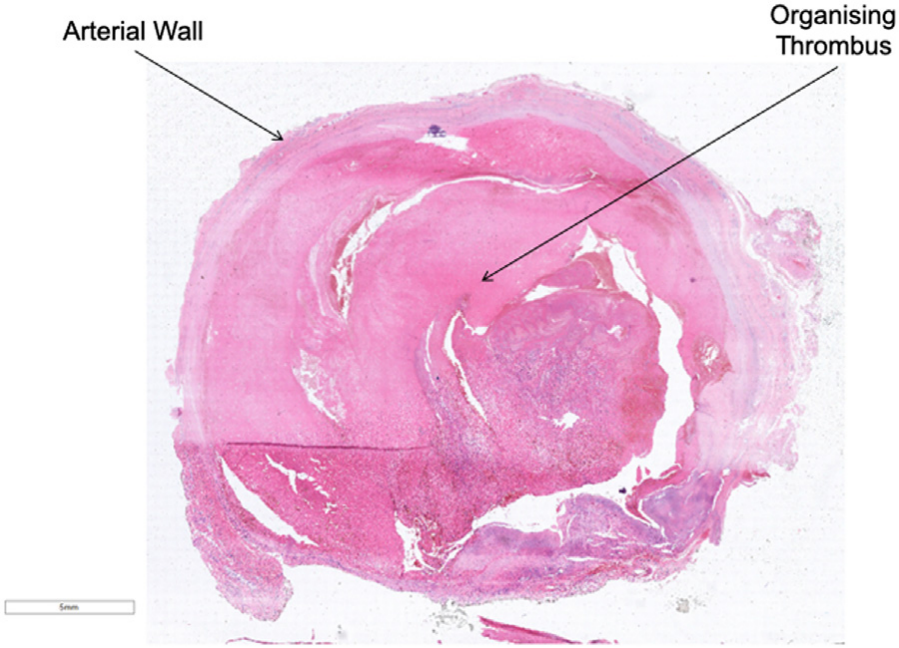
Histopathological features of the resected aneurysm. Hematoxylin and eosin (H&E) stain showing aneurysm with intact vessel wall and an organizing thrombus filling the vascular lumen.

To manage this patient, an excision of the aneurysm was performed after clapping the radial artery. The tissue was sent to histology laboratory. A vein graft was harvested from the left great saphenous vein (GSV) from below the knee to match the size of the radial artery. A microscopic anastomosis was performed using 8/0 prolene between the two ends of the radial artery and the vein graft after reversing the vein to dysfunction the venous valves. Blood flow was noted with adequate tension. Primary closure was performed for the overlying skin. The forearm was placed in splint for 10 days to prevent disruption to the anastomosis. The postoperative course was uneventful. The patient was discharged home on day 1. He was reviewed in the outpatient clinic with optimal outcomes (Fig. 3). Histological examination demonstrated intact vessel walls consistent with a true aneurysm (Fig. 4).

## Discussion

The published reports that describe aneurysms after AV ligation are sparse. A systematic review by Kordzadeh et al. identified 12 articles with 23 case reports of aneurysm developing after ligation; however, only one case reported the involvement of the radial artery.^5^ On further reviewing the original case by Marzelle et al., a radial artery aneurysm was reported in 33-year-old-man arising from a ligated radiocephalic fistula, which was initially presented with pain and swelling. The median time between the ligation of the AV fistula and the development of the aneurysm was 120 months after ligations and was managed with polytetrafluoroethylene (PTFE) bypass.^8^ Another case report by Malbecq et al. reported that the aneurysmal degeneration of the radial artery after ligation in a 55-year-old man presented with millimetric blue lesion on fingers. The median time between surgical ligation of AVF to diagnosis of arterial aneurysm was 70 months, and the aneurysm was managed medically with antiplatelet and anticoagulant therapy due to the anatomical variation of the palmar arch.^9^

It was evident that pain and swelling are the most commonly reported presentations of arterial aneurysms arising from AV fistula stump; however, it can also present with coldness, tingling, and/or rupture.^2^^,^^5^^,^^8^ Additionally, it was reported that median time between surgical ligation of AVF to diagnosis of arterial aneurysm ranged 6–280 months.^5^ In our case, it was 18 months from surgical ligation.

The reasons of AV fistula aneurysm after ligations are believed to stem from the pathophysiological changes that happen in the vessel wall after the creation of AV fistula, resulting in an increased in the shear forces and the activation of the free oxygen radicals which can enzymatically degrade collagen IV and V.^4^ We appreciate that the fistula developed 18 months after ligation, and thus this can be due to other factors such as the long-term use of corticosteroid and weakening impact this has on the remodelling process in the vessel wall due to the activation of the proinflammatory cytokines. Additionally, the steroid is linked to the development of the aortic aneurysm, and it is not a surprise that it may also causes aneurysms in other vessels. Other factors include the formation of a luminal thrombus which could be idiopathic or iatrogenic (previous dialysis or ligation surgery), long-term dialysis, and smoking, which lead to inflammation, medial wall thinning, and elastic tissue disassembly.^4^ Despite these predisposing factors, the reasons in our case remain unclear.

A wide range of diagnostic modalities is used, but duplex is considered the first modality of choice complimented by CT angiogram to visualize distal run-off. Other modalities have been described in some cases such as MRI to assist in surgical planning.^2^^,^^5^^,^^8^

Management options vary according to the presenting symptoms, site, size, and type of aneurysm. In most published cases, brachial artery aneurysm has been widely managed by surgical intervention by aneurysmectomy and arterial reconstruction using a synthetic or autologous venous graft.^1^, ^2^, ^3^, ^4^ Studies suggest that repair with autologous vein graft, such as the GSV interposition or brachial vein interposition, provides superior patency compared to the PTFE bypass and composite grafting.^5^ Interestingly, the management of this type of aneurysms with endovascular intervention has not yet been reported in any of the reported cases.

In conclusion, the aneurysmal dilation of the radial artery after AV fistula surgical ligation remains a very rare entity. It can present over a broad timeline from the time of fistula ligation. The most common presentation is a painful swelling. It is most commonly diagnosed with duplex scanning. The management options depend on multiple factors. Autologous venous interposition is preferred due to the high patency rate. Our case is the first case to highlight the use of the autologous vein graft in managing radial artery aneurysm arising from a ligated AV fistula.

## References

[1] Cox N, Sahnan K, Yee CP, Sritharan K. (2015 Jun 10). Brachial artery pseudoaneurysm arising from the stump of a ligated arteriovenous fistula. BMJ Case Rep.

[2] Dinoto E (2012 Nov). Giant brachial artery aneurysm following hemodialysis fistula ligation in a renal transplant patient: case report and literature review. Gen Thorac Cardiovasc Surg.

[3] Rodrigues R, Anacleto G, Lima P, Gonçalves A, Gonçalves Ó. (2019 Jan). Rupture of a true brachial artery aneurysm in a kidney transplant patient after arteriovenous fistula ligation: A rare presentation of an unusual disease. J Vasc Access.

[4] Anastasiadou C, Megalopoulos A, Tasiopoulou K, Intzos V. (2019 Jan). A Rare Case of Brachial Artery Aneurysm Following Hemodialysis Fistula Ligation in a Transplanted Patient. Vasc Endovascular Surg.

[5] Kordzadeh A, Barbara RMD, Ahmad AS, Hanif MA, Panayiotopoulos YP. (2015). Donor Artery Aneurysm Formation following the Ligation of Haemodialysis Arteriovenous Fistula: A Systematic Review and Case Reports. The Journal of Vascular Access.

[6] Monségu J., Bertrand B., Schiano P., Duriez P., Ollivier J.P. (2002). Radial artery occlusion after transradial artery coronary procedures: An ultrasonographic analysis. In AMERICAN JOURNAL OF CARDIOLOGY.

[7] Al-Zoubi N.A. (2018). Idiopathic true aneurysm of distal radial artery: case report. Vascular health and risk management.

[8] Marzelle J, Gashi V, Nguyen HD, Mouton A, Becquemin JP, Bourquelot P. (2012 Apr). Aneurysmal degeneration of the donor artery after vascular access. J Vasc Surg.

[9] Malbecq C, Hammer F, Pochet JM, Labriola L, Kanaan N, Devresse A, Lambert C, Mourad M, Snoeijs M, Darius T. (2021 Jul 29). Peripheral embolism as first and only clinical symptom of a true aneurysmal degeneration of the radial artery after ligation of a radiocephalic fistula. J Vasc Access.

